# Janus kinase inhibition for the treatment of refractory frontal fibrosing alopecia: A case series and review of the literature

**DOI:** 10.1016/j.jdcr.2023.07.037

**Published:** 2023-08-11

**Authors:** Charles Dunn, Victoria Griffith, Alexis Coican, Alexander Dane, William Chow, Savina Aneja, Rajiv Nathoo, Adam Leavitt, Spencer D. Hawkins

**Affiliations:** a Department of Dermatology, KCU – GME/ADCS Consortium, Maitland, Florida; b Department of Graduate Medical Education, Memorial Healthcare, Pembroke Pines, Florida; c Department of Graduate Medical Education, Orange Park Medical Center, Orange Park, Florida; d Advanced Dermatology & Cosmetic Surgery, East Greenwich, Rhode Island

**Keywords:** alopecia, baricitinib, frontal fibrosing alopecia, janus kinase inhibitors, lichen planopilaris, ruxolitinib

## Introduction

Frontal fibrosing alopecia (FFA) is a clinical variant of lichen planopilaris (LPP) characterized by slow, progressive, perifollicular inflammation with complete loss of hair and follicular ostia that primarily impacts the frontotemporal hairline.^1^ FFA is uncommon and impacts postmenopausal women disproportionately, although there are reports of affected men and children.^1^^,^^2^ The etiology of FFA is unclear, with hormonal factors, autoimmunity, genetic susceptibility, immune dysregulation, and environmental exposures hypothesized to play a role.^1^^,^^2^ In studies, the severity of LPP is often quantified on a scale of 1 (less severe) to 10 (more severe) using the lichen planopilaris activity index (LPPAI); alternatively, in LPP with FFA, the more specific frontal fibrosing alopecia severity index (FFASI) that accounts for hairline recession, inflammatory band, nonscalp loss, and associated features—quantified from 1 (less severe) to 100 (more severe)—is used.^3^^,^^4^ The disease can be debilitating, and largely because of a poor understanding of disease pathogenesis, currently, there is a lack of standardized evidence-based therapeutics for patients with FFA. Many recommendations are derived from findings in small case series or reports.^5^ Therapeutic options are often limited, responses to these treatments are variable, and complete remission is rare.^2^^,^^5^ Recent advances in translational science have revealed that the Janus kinase-signal transducer and activator of transcription pathway may play a significant role in lichenoid inflammatory diseases, including FFA/LPP.^6^ The advent of Janus kinase (JAK) inhibitors thus presents a mechanistically promising option for refractory cases, although limited literature has explored their therapeutic efficacy to date. Herein, we present 3 cases of patients diagnosed with refractory FFA/LPP who demonstrated significant improvement after treatment with targeted JAK inhibition, including 2 who were primarily receiving topical treatment and 1 who was provided a limited course of oral therapy. The present case series presents and reviews a promising novel treatment option to add to the therapeutic armamentarium for this challenging-to-treat condition.

## Case series

### Case 1

**Fig 1:**
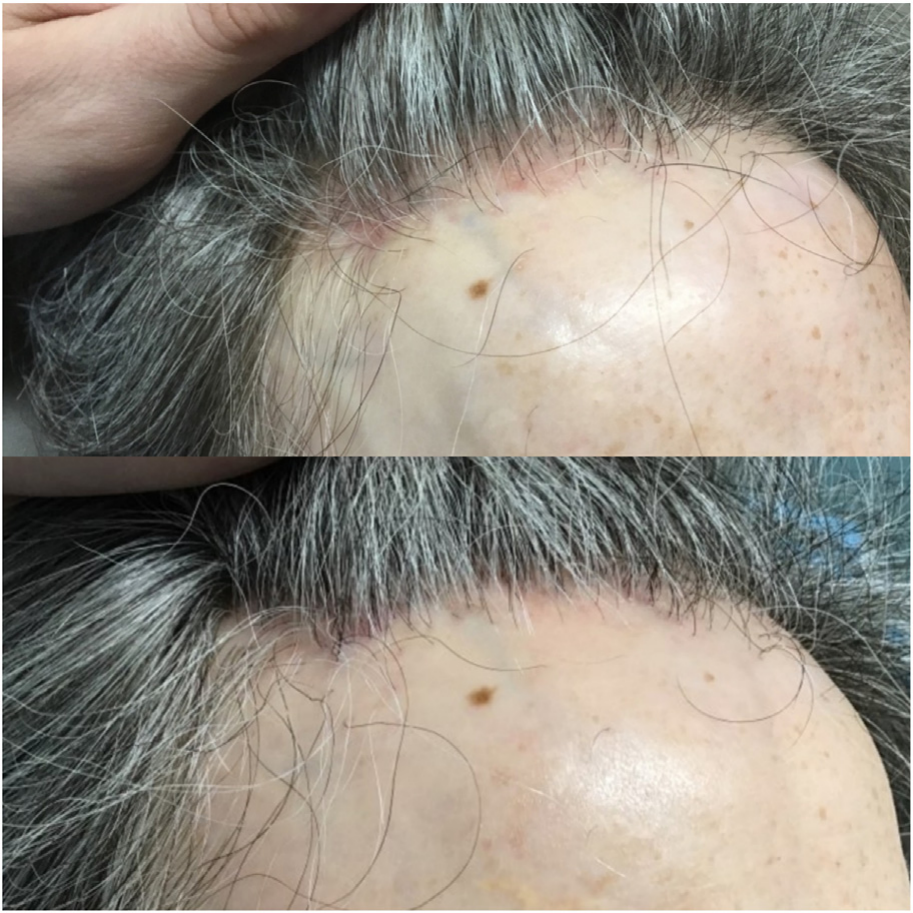
Frontal hairline of case 1 before (*top*) and 12 weeks after (*bottom*) the addition of topical ruxolitinib to her treatment regimen. Images demonstrate profound reduction of perifollicular erythema, absence of scale, and evidence of hair regrowth in previously affected areas.

A 47-year-old woman with a 4-year history of FFA presented to our clinic in December 2022 with concerns of progressive hair loss and scalp pruritus. She had been treated with escalating potencies of topical corticosteroids, monthly intralesional triamcinolone (5 mg/mL), topical pimecrolimus, oral dutasteride 0.5 mg, and daily oral minoxidil 1.25 mg for the preceding 18 months, with no sustained improvement. On examination, she demonstrated perifollicular erythema, prominent scale, focal areas of hair thinning from the frontal/temporal scalp, estimated frontal hairline recession of 2.8 cm, and lonely hairs showing absence of follicular openings, milky red areas, and blue-gray dots with dermatoscopic evaluation. Histopathologic evaluation at this time demonstrated a lichenoid interface dermatitis and lymphocytic infiltration at the follicular infundibulum, consistent with a diagnosis of LPP/FFA. Her estimated LPPAI score at that time was 7, and her FFASI score was 37. After discussing risks and benefits of alternative treatment options, she elected a trial of topical ruxolitinib 1.5%. Samples were provided to the patients with instructions to apply to the affected areas twice daily while maintaining the above oral treatments. All other topical treatments were discontinued while oral therapies were continued based on patient request. Follow-up evaluation at 12 weeks demonstrated drastic reduction in itch as well as significant improvement in perifollicular erythema and scale with an LPPAI score of 2 and FFASI score of 18 (Fig 1). Imaging evaluation also suggested frontal/temporal hair regrowth, with an estimated frontal hairline restoration of 1 cm. She continued with twice daily therapy for another 4 weeks before tapering to once daily dosing, with sustained absence of itch/erythema noted at 6-month follow-up in July 2023.

### Case 2

A 52-year-old woman presented to clinic in August 2019 with significant scalp pruritus. Her examination demonstrated frontal/temporal hairline recession of approximately 4 cm with perifollicular erythema and scale, loss of follicular ostia, and keratosis pilaris-like papules on the forehead. Biopsy confirmed cicatricial alopecia secondary to LPP with a clinical profile consistent with FFA (LPPAI: 8/FFASI: 41). She was started on nightly clobetasol 0.05% solution along with monthly intralesional triamcinolone (5 mg/mL) injections. Further, her symptoms progressed; therefore, oral finasteride 5 mg was added. Subsequently, after 6 months, finasteride was transitioned to daily oral dutasteride 0.5 mg and oral minoxidil 1.25 mg because of lack of efficacy. She continued these medicines for 15 months but experienced persistent issues with scalp pruritus and demonstrated clinical evidence of persistent disease activity with perifollicular erythema and scale on examination. Topical ruxolitinib 1.5% applied twice daily was added to her oral treatment regimen, and after 15 weeks of use, the patient endorsed complete resolution of itch with significant improvement in perifollicular erythema and scale at the hairline and temples (LPPAI: 3/FFASI: 22; Fig 2). She elected to discontinue oral treatments and demonstrated sustained clinical improvement at 6-month follow-up with topical ruxolitinib alone.

**Fig 2:**
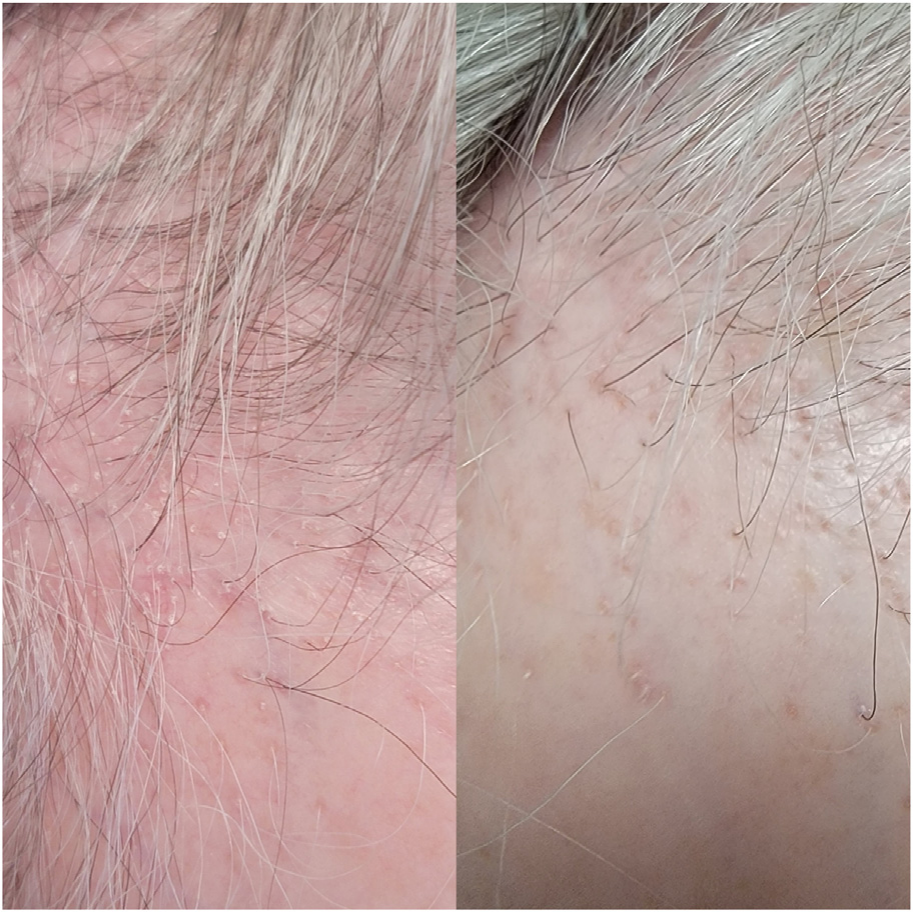
Side-by-side images of temporal hairline of case 2 before (*left*) and 4 weeks after (*right*) the application of topical ruxolitinib for frontal fibrosing alopecia. Images demonstrate significant reduction of perifollicular erythema and scale.

### Case 3

**Fig 3:**
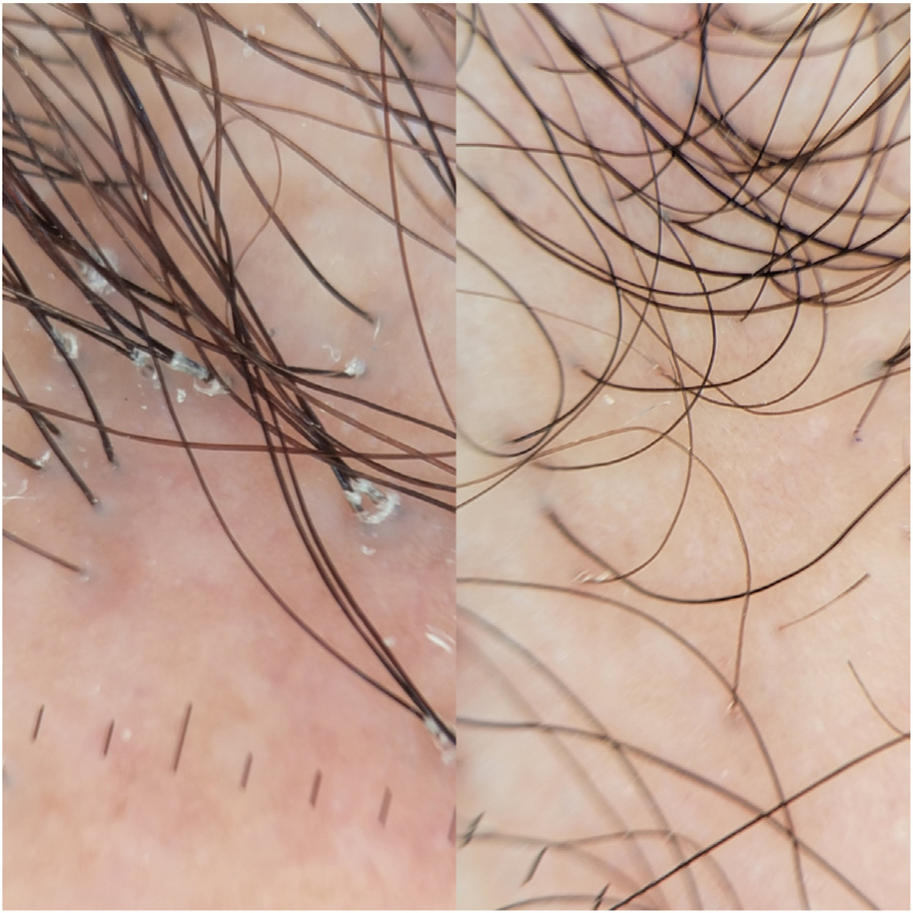
Trichoscopy image of case 3 showing frontal hairline before (*left*) and 4 weeks after (*right*) the addition of oral baricitinib/topical ruxolitinib to her therapeutic regimen. Significant reduction in apparent erythema and scale noted with patient-endorsed absence of pruritus.

A 42-year-old woman with no significant past medical history who was not receiving any chronic medications presented to our clinic with a 3-year history of hair loss. She noted frontal hairline thinning and moderate pruritus. On examination, she demonstrated frontal and temporal recession of 6.4 cm progressing behind her ears, flesh colored keratosis pilaris-like papules on her forehead, perifollicular scale, and erythema with loss of follicular ostium as well as a lonely hair sign. Her estimated LPPAI score was 7 and FFASI score was 54 at the time of initial evaluation. Her anterior neck demonstrated a hyperpigmented reticular patch with gray regression-like features on dermatoscopy. She was provided a clinical diagnosis of FFA with associated lichen planus pigmentosus and started on topical clobetasol solution twice daily for 2 weeks with alternative use of topical compounded tacrolimus 0.12% solution twice daily for 2 weeks. There was no improvement with the use of this regimen for 3 months. Oral dutasteride 0.5 mg daily, oral minoxidil 1.25 mg daily, oral doxycycline 100 mg daily, as needed, were then added for pruritus. After 6 months with no clinical improvement, intralesional triamcinolone (5 mg/mL) was additionally applied to affected areas every 4 weeks. Topical ruxolitinib 1.5% cream to be applied to her neck twice daily along with recommendations of strict photoprotection was added to address her neck findings. Further, after 6 months, she demonstrated modest improvement in her hyperpigmented neck rash, but there was no evidence of scalp improvement. She endorsed subjective progression of hair loss and consistent pruritus. Given the severity of the disease and lack of scalp improvement with 12 months of oral therapy and 6 months of intralesional corticosteroids, alternative treatment options were discussed, and the patient elected to pursue oral JAK inhibition. Baseline laboratory findings were obtained, and she was started on oral baricitinib 4 mg daily while continuing her other therapies. She demonstrated complete resolution of perifollicular scale/erythema, symptomatic improvement in pruritus, and absence of neck rash after 1 month of treatment, with an LPPAI score of 2 and FFASI score of 18. Baricitinib was discontinued after 2 months of use with no recurrence noted at 1 month follow-up (Fig 3).

## Discussion

FFA and LPP are lymphocytic variants of cicatricial alopecia that present rare clinical challenges because of their tendency for progression toward irreversible hair loss and the limited effectiveness of traditional therapies. Currently, there are no specific treatment guidelines for LPP/FFA, and the level of evidence for recommended therapies is weak.^6^ We present 3 cases to highlight the potential of JAK inhibitors as an innovative therapeutic avenue for these conditions. To date, there are limited case series suggesting a positive influence of JAK inhibition on LPP/FFA, with most studies focusing on oral treatments for recalcitrant cases of LPP (Table I). Shah et al^7^ demonstrated a clinically measurable improvement in LPP in 8 of 10 patients treated with oral tofacitinib, either as monotherapy or as adjunctive treatment. Moussa et al^3^ showed a baseline reduction in the LPPAI score of 5.8 in 12 patients with FFA/LPP treated with oral baricitinib. In a series of 9 patients with recalcitrant LPP treated with oral or topical tofacitinib, all patients demonstrated an initial positive response, with 2 of the 3 patients treated topically and 5 of 6 treated orally showing sustained efficacy.^8^ To our knowledge, our series appears to be the first to demonstrate the efficacy of topical ruxolitinib and adds to the growing body of literature supporting the role of baricitinib in recalcitrant cases of FFA/LPP.

**Table I:** Literature review of case series supporting the use of Janus kinase inhibition for lichen planopilaris/frontal fibrosing alopecia

Study	Disease variant	Patient No.	Oral or topical Janus kinase inhibitor	Average age (y)	Average disease duration (y)	Previous therapies	Average time to response	Concurrent therapies	Lichen planopilaris activity index improvement
*Yang (2018)*	LPP	*n* = 10	Oral Tofacitinib | 10-15 mg	55	4.1	TCS, ILK, topical minoxidil, excimer laser, doxycycline, HCQ, pioglitazone, finasteride, MPA	∗(Tx time 2-19 mo)	ILK, TCI, HCQ	−3.1
*Moussa (2022)*	FFA	*n* = 5	Oral Baricitinib | 3.4-6.8 mg	47.4	9.6	HCQ, TCS, ILK, TCI, PRP, finasteride, tofacitinib	2.5 mo	Dutasteride, spironolactone, minoxidil, finasteride	−1.87
	LPP	*n* = 7	Oral Baricitinib | 3.4-6.8 mg	43.6	8.3	HCQ, TCS, oral steroids, tofacitinib, bicalutamide	1 mo	TCS, minoxidil, ILK	−1.5
*Plante (2020)*	FFA	*n* = 1	Oral Tofacitinib | 5 mg	67	1	Dutasteride, naltrexone, TCI, pioglitazone,TCS	4 mo	Dutasteride, naltrexone, TCI, TCS	+2∗
		*n* = 1	Topical Tofacitinib | 2% cream	63	1	Finsteride, minoxidil, MPA, topical steroids	3 mo	Dutasteride, laser cap, minoxidil, MPA, naltrexone, TCI	+3∗
	LPP	*n* = 4	Oral Tofacitinib | 5 mg	52	2.5	Dutasteride, ILK, HCQ, TCI, pioglitazone, TCS	3.8 mo	Dutasteride, naltrexone, minoxidil, TCI, TCS	+2∗
		*n* = 2	Topical Tofacitinib | 2% cream	65	5	Doxycycline, dutasteride, HCQ, TCI, laser cap, TCS	4 mo	Dutasteride, ILK, TCI	−1∗
		*n* = 1	Combination: Oral Tofacitinib | 5 mg Topical Tofacitinib | 2% cream	59	6	Cyclosporine, dutasteride, excimer laser, HCQ, ILK, laser cap, MPA, TCI, prednisone, TCS	1 mo	Dutasteride, laser cap, naltrexone, TCI, TCS	+2∗
*Our case series*	FFA	*n* = 2	Topical Ruxolitinib | 1.5% cream	49.5	4	TCS, ILK, dutasteride, TCI	8.5 wk	Dutasteride, minoxidil	−5
		*n* = 1	Oral Baricitinib | 4 mg	42	3	TCS, TCI, dutasteride, minoxidil, doxycycline, ILK	4 wk	dutasteride, minoxidil, doxycyline, ILK	−7

*LPP*, Lichen planopilaris; *FFA*, frontal fibrosing alopecia; *HCQ*, hydroxychloroquine; *MPA*, mycophenolic acid; *ILK*, intralesional triamcinolone; *TCS*, topical corticosteroids; *TCI*, topical calcineurin inhibitor; *PRP*, platelet rich plasma.

∗ Graded for baseline severity from −3 (much worse) to +3 (greatly improved).

Recent mouse model studies suggest that interferon (IFN) signaling plays a profound role in the pathogenesis of LPP.^7^ Increased expression of IFN-inducible chemokines, including interleukin (IL)-2, IL-6, IL-7, IL-11, IL-13, IL-15, and IFN-gamma at the level of the follicular bulge have been demonstrated in lesional skin.^9^ This results in the recruitment of cytotoxic T-cells, decreased expression of transforming growth factor (TGF)-beta, and ultimate loss of hair follicle immune privilege. Additionally, patients with LPP have been shown to have significantly upregulated expression of JAK 1 and 3 in dermal inflammatory cells—a profile that supports the role of inhibition as a therapeutic option.^2^

JAK inhibitors have been gaining attention as a class of inhibitors in the field of dermatology for their impressive immunomodulatory effects and variety of effected clinical pathways. They have demonstrated recent efficacy in the off-label treatment of various lichenoid dermatoses.^7^ Ruxolitinib and baricitinib are relatively rare as they are both potent selective inhibitors of JAK1 and JAK2—arms of the Janus kinase-signal transducer and activator of transcription pathway known to drive IFN-mediated inflammation.^10^ While further immunologic and clinical analyses are necessary to fully understand the precise mechanism by which JAK inhibitors exert their therapeutic effect in FFA/LPP, this case series supports the critical role of JAK1/JAK2 in the pathogenesis and potential treatment.

The use of JAK inhibitors in the presented cases, both topically and orally, resulted in remarkable clinical improvement in subjective and objective findings. This was particularly evident in the first and second cases, where topical ruxolitinib led to the resolution of pruritus, perifollicular erythema, and scale. An anecdote suggests that topical therapies work better for FFA than for generalized LPP, with authors hypothesizing that thinner skin of the frontal scalp allows topical preparations to penetrate to the level of the follicular bulge more effectively than on thicker areas of the scalp. From the perspective of patients, it should be noted that although creams tend to be more efficacious than solutions or other vehicles for JAKs, their use may be cumbersome for some patients to apply to larger areas of the scalp, leading to decreased compliance.

Evaluation of images from case 1 suggested hair regrowth after treatment, potentially implying that treatment with JAK inhibitors may not only halt disease progression but could also contribute to follicular recovery, although it should be noted that this patient received concurrent oral minoxidil and dutasteride to promote hair growth, confounding interpretation of these results. This finding supports the theory that JAK inhibition could restore follicular immune privilege, and if started early enough, it could potentially reverse the disease process—a discovery that could alter the fundamental therapeutic ladder for this condition toward favoring JAK inhibition earlier in the treatment course—although this would require further studies. Case 3 is also notable as the patient presented with comorbid lichen planus pigmentosus—a known associated entity with FFA that often proves refractory to standard therapies for FFA and requires its own treatment profile.^10^ The fact that both conditions responded positively to JAK inhibition supports a common etiologic pathway and adds to the theory that JAK inhibitors can provide a robust response in cases of FFA/LPP refractory to conventional treatments.

Despite the exciting prospect of a promising treatment option, our case series has limitations inherent to its design. Oral treatments were continued during the use of JAK inhibition in 2 cases; therefore, it is possible our findings represent natural disease stagnation or delayed benefit from these oral therapies instead of actual therapeutic efficacy with JAK inhibitors. However, the timeline in all cases with sustained lack of benefit using the combination of oral and traditional topical therapies, followed by drastic improvement within 12 weeks of the addition of JAK inhibitors, strongly supports, at minimum, the adjunctive therapeutic benefit of these medicines in cases of FFA. The follow-up period was also relatively short, and longer observation periods are truly required to determine hair growth/progression patterns and whether these effects are maintained. A larger sample size and a more diverse patient population would also provide a more robust validation of our findings. Additionally, although the clinical profile of case 3 was clearly consistent with FFA/LPP, the lack of histopathologic confirmation limits the full conclusions of this patient. Randomized controlled trials are needed to confirm these results and to determine optimal dosing and duration of treatment with JAK inhibitors.

In summary, this case series highlights the therapeutic potential of targeted JAK inhibitors, particularly topical ruxolitinib and oral baricitinib, in the treatment of specific patients with recalcitrant FFA/LPP. Further research is needed to assess the safety and efficacy of these medicines in a larger cohort of patients and to elucidate their precise mechanisms of action in treating FFA/LPP. However, this case series could pave the way for the development of new treatment options for this condition.

## Conflicts of interest

None disclosed.
